# B Cell Receptor Affinity for Insulin Dictates Autoantigen Acquisition and B Cell Functionality in Autoimmune Diabetes

**DOI:** 10.3390/jcm5110098

**Published:** 2016-11-08

**Authors:** Thomas A. Packard, Mia J. Smith, Francis J. Conrad, Sara A. Johnson, Andrew Getahun, Robin S. Lindsay, Rochelle M. Hinman, Rachel S. Friedman, James W. Thomas, John C. Cambier

**Affiliations:** 1 Department of Immunology and Microbiology, University of Colorado School of Medicine, Aurora, CO 80045, USA; thomas.packard@ucdenver.edu (T.A.P.); mia.smith@ucdenver.edu (M.J.S.); frank@coloradogreenlab.com (F.J.C.); johnson.sara@comcast.net (S.A.J.); Andrew.Getahun@ucdenver.edu (A.G.); robin.s.lindsay@ucdenver.edu (R.S.L.); rhinman99@gmail.com (R.M.H.); FriedmanR@NJHealth.org (R.S.F.); 2 Department of Biomedical Research, National Jewish Health, Denver, CO 80206, USA; 3 Department of Microbiology, Immunology and Pathology, Colorado State University, Fort Collins, CO 80523, USA; 4 Division of Rheumatology and Immunology, Vanderbilt University Medical Center, Nashville, TN 37232, USA; james.w.thomas@vanderbilt.edu

**Keywords:** BCR, B cell antigen receptor, insulin, type 1 diabetes (T1D), non-obese diabetic mouse (NOD)

## Abstract

B cells have been strongly implicated in the development of human type 1 diabetes and are required for disease in the NOD mouse model. These functions are dependent on B cell antigen receptor (BCR) specificity and expression of MHC, implicating linked autoantigen recognition and presentation to effector T cells. BCR-antigen affinity requirements for participation in disease are unclear. We hypothesized that BCR affinity for the autoantigen insulin differentially affects lymphocyte functionality, including tolerance modality and the ability to acquire and become activated in the diabetogenic environment. Using combined transgenic and retrogenic heavy and light chain to create multiple insulin-binding BCRs, we demonstrate that affinity for insulin is a critical determinant of the function of these autoreactive cells. We show that both BCR affinity for insulin and genetic background affect tolerance induction in immature B cells. We also find new evidence that may explain the enigmatic ability of B cells expressing 125 anti-insulin BCR to support development of TID in NOD mice despite a reported affinity beneath requirements for binding insulin at in vivo concentrations. We report that when expressed as an antigen receptor the affinity of 125 is much higher than determined by measurements of the soluble form. Finally, we show that in vivo acquisition of insulin requires both sufficient BCR affinity and permissive host/tissue environment. We propose that a confluence of BCR affinity, pancreas environment, and B cell tolerance-regulating genes in the NOD animal allows acquisition of insulin and autoimmunity.

## 1. Introduction

Type 1 diabetes (T1D) is an autoimmune disease in which an inappropriate adaptive immune response drives destruction of the beta cells in the pancreas, resulting in lifelong insulin dependence. Though it is generally accepted that the effector cells that ultimately mediate pathology are T lymphocytes, critical early processes are likely dependent on B cells. In a clinical trial, B cell depletion in new onset-T1D patients resulted in preserved pancreas function [1]. As a consequence of B cell activation, serum anti-insulin antibodies are found in T1D patients; often appearing before clinical onset, and when found in conjunction with other islet-reactive antibodies, they are a good biomarker of disease risk [2]. Though the occurrence of these anti-islet antibodies is associated with diabetes, and there is evidence for a role of maternal immunoglobulin as promoters of disease in mice, this may not extend to human T1D; other studies have shown that B cells lacking the capacity to secrete antibody can support the development of diabetes [3,4,5,6].

Recent examination of pancreas tissue from new-onset T1D patients revealed that B cell infiltration correlates to the rate and extent of beta cell destruction [7]. In short, a study by Leete et al. showed that complete islet destruction early in life was associated with extensive B cell infiltration of the pancreas, and that incomplete islet destruction and delayed onset (>13 years old) occurred in individuals with low B cell insulitis. Together, these studies of human T1D highlight B cells as key players in the development of disease.

What could be the pathogenic function of B cells in T1D? In the non-obese diabetic (NOD) mouse model, B cells are required for the development of diabetes [8,9,10]. This functionality is dependent on B cell antigen receptor (BCR) specificity, as transgenic restriction to non-autoreactive BCR (e.g., VH281, IgHEL) protects NOD mice from disease [11,12]. Insulin-specific B cells fulfill this requirement for diabetes, though little is known regarding the affinity of this BCR-antigen (Ag) interaction. Previous studies using the transgenic insulin-binding BCR 125Tg have shown that these animals develop disease similar to non-transgenic NOD [13]. However, mice expressing the 125 heavy chain transgene (VH125) paired with endogenous light chain develop diabetes at a significantly higher rate and penetrance [11,13]. We hypothesize that the affinity of insulin binding by the BCR somehow underlies this difference in disease development.

It is likely that autoreactive B and T cells collaborate to drive the development of diabetes. Linked recognition of cognate self-Ag would proceed with the B cell acquiring insulin and becoming activated, followed by processing and presentation of insulin peptides on MHC, ultimately driving activation of insulin-specific T cells. Previous investigations have demonstrated a requirement for MHC class II and class I expression by B cells for NOD disease, underscoring their role in Ag presentation and cross-presentation to CD4 and CD8 T cells—which proceed to mediate islet destruction [14,15]. The affinity of the BCR–insulin interaction is likely critical to this outcome. It affects the acquisition of Ag: i.e., the likelihood that a BCR will interact with Ag, as well as antigen receptor signaling and cell activation. The plasma concentration of hormonal insulin is quite low, at sub-nanomolar levels [16,17]. Compounded with competition with insulin receptors, a low-affinity BCR would likely not bind insulin [18]. Therefore, B cells bearing BCR with low-affinity anti-insulin would be predicted to be Ag “ignorant” in the animal: characterized by a lack of BCR-bound Ag (receptor occupancy). Even a strong BCR interaction with circulating insulin is unlikely to induce BCR signaling due to the monovalent status of the hormone in blood [16,19]. Thus it is unclear how B cell participation in T1D is initiated. It seems likely that in T1D-prone mice conditions must exist that lead to exposure of high-affinity insulin-binding B cells to multimeric insulin.

Interestingly, studies of mAb125—the origin of the 125Tg animal—characterized this Ig molecule as having a relatively low affinity for insulin. Estimates of the K_D_ for mAb125 range from 5–8 × 10^−6^ M for rodent insulin to 3 × 10^−8^ M for human insulin [13,20,21,22,23]. In light of this micromolar affinity for a sub-nanomolar Ag, early studies of 125Tg mice were surprising in terms of the detection of significant receptor occupancy in vivo and the ability of this transgenic BCR to drive disease development [13]. Even in non-diabetes-susceptible B6 animals, these 125Tg B cells were receptor-occupied and displayed a phenotype similar to anergy [13,22,24]. Anergy is a reversible process dependent on chronic BCR signaling, the requirements for which should not be supported by a low-affinity BCR binding to a pulsatile monovalent Ag. Though the investigators pointed out the discordance in outcomes predicted by affinity versus effects observed in vivo, the underlying cause was unknown.

As mentioned above, 125Tg NOD animals have less disease than the VH125 heavy-chain-only transgenics. In VH125 B cells, endogenous light chains can enable insulin binding with varied affinity [25,26]. Thus, we investigated the affinity of the insulin-binding BCR as a determinant of B cell function by varying light chain usage. We hypothesized that affinity for insulin would affect the tolerance modality engaged: in vivo, high-affinity cells would bind insulin and potentially be deleted or anergized, whereas low-affinity cells would remain ignorant. Additionally, we hypothesized that BCR affinity for insulin affects the B cell’s ability to drive diabetogenic processes, most fundamentally at the level of acquisition of antigen to enable subsequent T cell collaboration.

For non-self immunity, affinity is positively correlated with functionality [27,28]. However, this paradigm is not necessarily true for autoimmunity, as high-affinity binding to self may increase tolerance enforcement [29]. Thus, our study sought to investigate the functionality of insulin-specific BCR-binding affinity, in both tolerogenic and potentially pathogenic settings. We used a novel approach, combining transgenic heavy chain with retrogenic light chains to form the BCR (Transgenic-Retrogenic or TR-B cells). Our results show that the 125 Ig has significantly higher binding to insulin when expressed as a BCR than predicted by studies of its soluble form, which may explain the aforementioned discrepancy of detectable BCR occupancy in 125Tg animals. We find that affinity for insulin significantly affects the engagement of tolerance programs in vitro, and that these tolerant cells are less potent Ag presenting cells. Finally, we show that, in vivo, both the host environment and BCR affinity for insulin are determinants for acquisition of autoantigen.

## 2. Experimental Section

### 2.1. Animals

NOD and C57BL/6 animals were obtained from the Jackson Laboratory (Bar Harbor, ME, USA). Dr. J.W. Thomas (Vanderbilt) generously provided NOD.VH125, NOD.VH281, B6.VH125, and B6.VH281 animals. The NOD.VH125 were crossed to NOD.RAG−/− (NOD.129S7(B6)-Rag1^tm1Mom/J^; Jackson, Bar Harbor, ME, USA), and B6.VH125 crossed to B6.RAG2−/− (Rag2^tm1.1Cgn^; Jackson, Bar Harbor, ME, USA). B6 animals were crossed to H2^g7^.B6 (Jackson). Female animals were between eight and 12 weeks of age at the time of experiments. These animals were bred and housed in the specific pathogen-free facility at the National Jewish Health Biological Resource Facility. All experimental use was subject to, and in accordance with, prior approval by the National Jewish Institutional Animal Care and Use Committee.

### 2.2. Recombinant Immunoglobulin Expression

Plasmid DNA encoding the template for VL125 and VLEW6 was obtained from Dr. J.W. Thomas (Vanderbilt) [25]. An additional light chain, VLA12, was cloned from a hybridoma generated by immunization of three female NOD.VH125 littermates with bovine insulin (Sigma, St. Louis, MO, USA) in IFA. Splenocytes from these animals were isolated, pooled, and fused to myeloma partner cells with polyethylene glycol, as per standard techniques [30]. Hybridomas were screened for insulin binding by ELISA. Bovine insulin (Sigma, St. Louis, MO, USA) capture antigen was bound to 96-well ELISA (Nunc-Thermo Fisher Scientific, Waltham, MA, USA) plates via adsorption and blocked with solution of PBS/milk fat. Hybridoma supernatants were added to blocked bovine insulin-coated wells and washed with PBS-Tween (0.05%; Sigma, St. Louis, MO, USA), followed by incubation with HRP-conjugated polyclonal secondary antibody (Southern Biotech, Birmingham, AL, USA); then they were washed again, detected by addition of colorimetric HRP substrate (TMB; Sigma, St. Louis, MO, USA), stopped by addition of 1N aqueous sulfuric acid (Thermo Fisher Scientific, Waltham, MA, USA), and absorbance measured at 450 nm by an SpectraMax absorbance microplate reader (Molecular Devices, Sunnyvale, CA, USA).

For expression of anti-insulin antibodies, VL genes were amplified by PCR and ligated into an expression vector containing the human kappa constant region, and VH125 was cloned into an expression vector containing the human γ1 heavy-chain constant region, as previously described [31]. Briefly, Human Embryonic Kidney (HEK) 293 cells were maintained in DMEM (Gibco-Thermo Fisher Scientific, Waltham, MA, USA) supplemented with 2 mM l-Glutamine (Gibco-Thermo Fisher Scientific, Waltham, MA, USA), 100 Units/mL Penicillin, 100 µg/mL Streptomycin (Life Technologies, Carlsbad, CA, USA), 0.05 mM β-mercaptoethanol (Sigma, St. Louis, MO, USA), and 10% heat-inactivated FBS (Atlas Biologicals, Ft. Collins, CO, USA) that had been pre-adsorbed with Protein G to remove bovine IgG. The heavy- and light-chain plasmids were co-transfected into HEK 293 cells at 1:1 molar ratio, using Effectene Transfection Reagent (Qiagen, Valencia, CA, USA) according to the manufacturer’s protocols. Five days post-transfection, the supernatants were removed and centrifuged at 2000 RCF and 0.22 μM filtered. Clarified supernatant was incubated with Protein G-Sepharose beads (GE Healthcare Life Sciences, Pittsburgh, PA, USA) overnight at 4 °C. Protein-G beads were pelleted by 200 RCF centrifugation, and loaded onto a chromatography column (Bio-Rad, Hercules, CA, USA). Bound Ig was eluted from the beads using a 100 mM pH 1.9 glycine-HCl buffer. Eluted Ig was buffer-exchanged into PBS via 50 kD centrifugal filter unit (Amicon-EMD Millipore, Billerica, MA, USA). Purified Ig was analyzed by SDS-PAGE and Coomassie staining, and the concentration was determined by OD280 absorbance using a NanoDrop (Thermo Fisher Scientific, Waltham, MA, USA) programmed for IgG molecular weight (150 kDa) and extinction coefficient (5800 M^−1^·cm^−1^).

### 2.3. Surface Plasmon Resonance Analysis of Antibody Affinity

Affinity analysis was performed using a BIAcore 2000 instrument (GE Healthcare, Chicago, IL, USA). Purified Igs were conjugated to individual flow cells of carboxymethylated dextran-coated CM-5 Sensor Chips (GE Healthcare, Chicago, IL, USA) according to the manufacturer’s protocols. Briefly, the carboxymethyl dextran surface of the flow cell was activated by injection of aqueous 0.4 M 1-ethyl-3-(3-dimethylaminopropyl)-carbodiimide (EDC; Sigma, St. Louis, MO, USA) and 0.1 M *N*-hydroxysuccinimide (NHS; Sigma, St. Louis, MO, USA) at a flow rate of 10 µL/min for 7 min. Following activation, recombinant Ig prepared as above in PBS (pH 7.2) was injected at a concentration of 25 µg/mL, at a flow rate of 10 µL/min for 7 min. Following ligand binding, 1 M ethanolamine-HCl (pH 8.5; Sigma, St. Louis, MO, USA) was injected at a flow rate of 10 µL/min for 7 min to quench excess succinimide esters on the chip surface. Following covalent binding of Ig to the chip, sequential injections of PBS (pH 7.2), regeneration buffer (10 mM glycine, pH 2.0), followed by PBS (pH 7.2) were performed to pre-wash the bound Ig. The Ig immobilization was monitored and confirmed by measurement of response units (RU) throughout the process.

Analysis of soluble insulin-binding kinetics was performed as previously described [32]. Briefly, human insulin (Sigma, St. Louis, MO, USA) was dissolved in PBS (pH 7.2), centrifuged at 18,000 RCF to remove aggregates, and dilutions prepared. Kinetics of binding to immobilized Ig were analyzed following injection of multiple concentrations of insulin (100 nM–100 μM in PBS) at a flow rate of 10 µL/min for 2 min, followed by injection of PBS (pH 7.2) for 5 min, then washed with regeneration buffer as above to remove remaining bound insulin. Association kinetics were assessed for 30 s beginning 10 s after insulin injection. Dissociation kinetics were assessed for one minute, beginning 20 s after PBS injection. Analyses were performed using BIAevaluation software (GE Healthcare, Chicago, IL, USA): kinetic rates were calculated based on the insulin concentration and fitted to a Langmuir isotherm model; aggregate *K*_D_s are reported. Normalization for visualization of response curves included background subtraction, setting time zero at insulin injection, and removal of outlier spike points that occur during injection changes.

### 2.4. B Cell Differentiation in Culture

Bone marrow was harvested from heavy-chain transgenic, NOD.RAG−/−.VH125, or B6.RAG2−/−.VH125 female donor animals. Following red cell depletion, single-cell suspensions were filtered through a 40 μm screen and 6.5 × 10^6^ cells were plated in 0.5 mL B cell differentiation medium/well in 24-well plates (Falcon-Thermo Fisher Scientific, Waltham, MA, USA). B cell differentiation medium was prepared using IMDM (Life Technologies, Carlsbad, CA, USA) supplemented with 2 mM l-Glutamine (Gibco-Thermo Fisher Scientific, Waltham, MA, USA), 100 Units/mL Penicillin, 100 µg/mL Streptomycin (Life Technologies, Carlsbad, CA, USA), 0.05 mM β-mercaptoethanol (Sigma, St. Louis, MO, USA), 10% heat-inactivated FBS (Atlas Biologicals, Ft. Collins, CO, USA), and 50–100 U/mL IL-7, as previously described [33]. Bone marrow cells were cultured 18–48 h before retroviral infection and harvested at least 48 h after infection.

### 2.5. Retroviral Expression of Immunoglobulin Light Chains

Chimeric VL-human Igκ constant genes from the above-described VL125, VLA12, or VLEW6 expression vectors were cloned into MSCV-IRES-GFP by PCR amplification and directional restriction as previously described [34]. Briefly, primers were designed for PCR amplification of template from the above light-chain expression vectors. For directional cloning, a 5′ primer was designed with a BglII site before the Kozak sequence and 3′ with SalI after the stop codon: Forward 5′-AATAGATCTGCCACCATGGGATGGTCATGTATCATCC-3′, Reverse 5′-TATGTCGACCTAACACTCTCCCCTGTTGAAGCTC-3′. PCR products and empty vector plasmid were incubated with BglII and SalI restriction enzymes (New England Biolabs, Ipswich, MA, USA) according to the manufacturer’s protocols. The digested DNA was gel purified and ligated in multiple ratios of vector:insert with T4 ligase (Life Technologies, Carlsbad, CA, USA) overnight. Ligation mixtures were used to transform Stbl2 competent cells (Life Technologies, Carlsbad, CA, USA) per the manufacturer’s protocols, and transformed cells were plated overnight on lysogeny broth (LB) agar plates + 50 μg/mL ampicillin (Sigma, St. Louis, MO, USA) at 30 °C. Single colonies were picked and grown overnight in LB + 50 μg/mL ampicillin at 30 °C. Plasmids were purified from overnight cultures using Miniprep (Qiagen, Valencia, CA, USA) and insert verified by restriction enzyme digestion, followed by sequencing. Infectious retrovirus was packaged in Phoenix Eco cells [35] by co-transfection with light-chain-MSCV vector + pCLEco in a 5:1 mass ratio [36]. Supernatant was harvested at 48 and 72 h post-transfection and used fresh, or flash frozen and stored at −80 °C. Viral supernatant was used to spin-infect IL-7 cultured pre-B cells, prepared as above, with slight modifications of the previously described methods [37]. Briefly, cells previously plated in 24-well plates were spin-infected with 1–2 mL/well of supernatant (with IL-7 & polybrene) at 900 RCF for 2 h at 37 °C. Transduction was assessed based on expression of GFP and/or human Igκ staining by flow cytometry >48 h post-infection.

### 2.6. Analysis of Insulin Binding by TR-B Cells

Immature TR-B cells were prepared by transduction of NOD. RAG−/−.VH125 or B6.RAG2−/−.VH125 heavy-chain transgenic B cells with retroviruses encoding light chains as described above. The resultant TR-B cells are noted within as NOD or B6, depending on bone marrow donor background, and designated by the VL retrovirus (e.g., NOD. RAG−/−.VH125 infected with VLA12-MSCV-GFP results in a NOD A12 TR-B cell). For non-functional studies, such as binding to labeled insulin, cells were harvested 3–5 days after transduction. Biotin-insulin was prepared per the manufacturer’s protocols using recombinant human insulin (Sigma, St. Louis, MO, USA) labeled with EZ-Link Sulfo-NHS-Biotin (Thermo Fisher Scientific, Waltham, MA, USA) at the relative molar concentrations needed to achieve an average of 1 biotin label per insulin molecule. For cell staining this biotinylated insulin was added to a final concentration of 1 μg/mL, unless otherwise indicated. Concurrent with the addition of insulin-biotin, cells were labeled with an antibody panel usually consisting of APC-eFluor780-anti-B220 (0.5 μg/mL; eBiosciences, San Diego, CA, USA), Alexa647-Fab-anti-IgM (b-7-6, prepared in-house), and PerCP-Cy5.5-anti-human kappa (5 μL per test; BD Biosciences, San Jose, CA, USA). After incubation of 30–60 min on ice, cells were washed and incubated with Brilliant Violet 421-streptavidin (0.2 μg/mL; BioLegend, San Diego, CA, USA) for 15–30 min on ice, followed by washing and analysis by flow cytometry.

### 2.7. Analysis of Insulin-Binding B Cells in VH125 NOD Animals

Cells were isolated from the spleen, non-draining inguinal lymph nodes, and pancreatic lymph nodes of 6–8-week-old non-diabetic (blood glucose <120 mg/dL) and 12–16-week pre-diabetic (blood glucose 180–250 mg/dL) NOD.VH125 female mice. We define mice with a blood glucose level of 180–250 mg/dL as “pre-diabetic” in our colony based on longitudinal studies of blood glucose measurements starting at eight weeks of age in our NOD.VH125. We have found that while individual readings vary on a day-to-day basis, once blood glucose reaches 180 mg/dL, overt diabetes occurs within one week in the majority (90%) of these mice. Cells were stained in PBS containing 1% BSA and 0.02% sodium azide with insulin-biotin and PE-anti-B220 (BD Biosciences, San Jose, CA, USA) for 30 min. Cells were washed and then incubated with Brilliant Violet 421-streptavidin (BioLegend, San Diego, CA, USA) or 15 min, followed by washing and analysis by flow cytometry.

### 2.8. Flow Cytometric Analysis & Cell Sorting

Following manipulation and staining described above, cells were analyzed using an LSR II or LSR Fortessa (BD Biosciences, San Jose, CA, USA). Cell sorting was performed using MoFlo XDP (Beckman Coulter, Brea, CA, USA), iCyt Synergy (Sony Biotechnology, San Jose, CA, USA), or FACSAriaII (BD Biosciences, San Jose, CA, USA). Analyses of flow data were performed using FlowJo software, versions 7 through 10 (FlowJo, LLC, Ashland, OR, USA).

### 2.9. Analysis of Intracellular Free Calcium Concentration, [Ca^2+^]_i_

Cells in culture were equilibrated to the ambient laboratory temperature and loaded with Indo-1 AM at a concentration of 1 μM (Sigma, St. Louis, MO, USA), as previously described, with slight modifications [38]. During loading, cells were stained with APC-eFluor780-anti-B220 (0.5 μg/mL; eBiosciences, San Diego, CA, USA) and Alexa647-anti-IgM (f(ab) b76, prepared in-house). Stimuli were diluted such that the addition of 10 µL would result in a stimulating concentration of 5 µg/mL of polyclonal goat anti-mouse IgM F(ab’)_2_ (Jackson ImmunoResearch, West Grove, PA, USA), or 50 µg/mL of human insulin (Sigma, St. Louis, MO, USA). Relative intracellular calcium [Ca^2+^]_i_ was measured by the fluorescence ratio of the calcium bound/unbound Indo ratio (390 nm/490 nm emission). FlowJo software (FlowJo LLC) was used to assess kinetics, as well as integrate the area under the curve (AUC) and the peak height over baseline.

### 2.10. Measurement of BCR Occupancy by Insulin

For quantitation of BCR occupancy, immature B cells prepared as described above were treated with soluble unlabeled human insulin (Sigma, St. Louis, MO, USA) at concentrations indicated in the figures and figure legends. These cells were then subjected to a “quick wash” (10× volume PBS added, followed by centrifugation for 20 s at 10,000 RCF; repeat). After this wash, biotin-mAb123 (1 μg/mL final) was added to the cell suspension along with other staining Abs, followed by incubation for 30–60 min on ice. The mAb123 recognizes an insulin epitope that is distinct from, and not sterically hindered by, the 125 antibody [20,22,39]. Biotin-mAb123 was prepared by Protein-G purification as described above from supernatant of the 123 hybridoma, a gift from Dr. J.W. Thomas (Vanderbilt UMC, Nashville, TN, USA), and conjugated to NHS-biotin (Pierce-Thermo Fisher Scientific, Waltham, MA, USA) as described above. Cells were then quick-washed, and bound biotin-mAb123 revealed using Brilliant Violet 421-streptavidin (0.2 μg/mL; BioLegend, San Diego, CA, USA). For ex vivo analyses, mAb123 was added to a suspension of single cells harvested from tissue following a quick wash, as above, but exogenous insulin was omitted.

### 2.11. Preparation of Mutant Ig Light Chains

The non-insulin-binding light-chain mutant VLYF was created by site-directed mutagenesis, replacing the VLA12 CDR3 p3 tyrosine with phenylalanine. VLA12 pMSCV, described above, was used as the template. QuikChange^®^ Lightning (Agilent Technologies, Santa Clara, CA, USA) was used in accordance with to the manufacturer’s directions, using primers designed by the QuikChange^®^ Primer Design program (Agilent Technologies, Santa Clara, CA, USA). Mutant plasmid was used to transform Stbl2 competent cells (Life Technologies, Carlsbad, CA, USA) and single colonies were grown overnight in lysogeny broth + 50 μg/mL ampicillin (Sigma, St. Louis, MO, USA) at 30 °C. Plasmids were purified using Miniprep (Qiagen, Valencia, CA, USA) and mutation verified by sequencing, for in-frame mutation, and subsequently utilized to construct TR-B cells as described above, confirming functional expression by staining human kappa constant region.

### 2.12. In Vitro Induction of B Cell Tolerance

B cell tolerance was induced as previously described [22]. Briefly, TR-B cells were prepared as above. IL-7 was removed from culture at day 5, and recombinant human insulin (Sigma) or 5 μg/mL goat-anti-mouse Ig f(Ab’)_2_ (GAMIM; Jackson ImmunoResearch, West Grove, PA, USA) was added at the indicated concentrations. Cells were harvested at the times indicated. For non-calcium mobilization experiments, cells were cooled on wet ice, washed, and stained with a cocktail of labeling Abs as described above, with slight modification. To measure activation, PE-Cy7-anti-CD86 (2 μg/mL; BioLegend, San Diego, CA, USA) was added, and PerCP-Cy5.5-anti-CD69 (2 μg/mL; BioLegend, San Diego, CA, USA) replaced human kappa staining in some panels.

### 2.13. Adoptive Transfer of B Cells

Female NOD.VH281 or B6.H2^g7^.VH281 host animals were sublethally irradiated (400 rads) 4–18 h before transfer. TR-B cells were prepared as described above and sorted for expression of GFP. Although there was some variation among experiments, we transferred the same number of TR-B cells per mouse within each experiment. Transfers varied from 5 × 10^5^ to 2 × 10^6^ injected TR-B cells per mouse. Sorted cells were maintained on ice, resuspended in 100 μL of PBS and injected via the tail vein.

### 2.14. Multiphoton Microscopy

Pancreatic lymph nodes were harvested from NOD.VH281 recipients of TR-B cells one week post-transfer, or recipients of TR-B cells one week post-transfer and insulin-specific T cells one day post-transfer. Freshly isolated PLNs were immobilized on coverslips using Vetbond Tissue Adhesive (3M, Minneapolis, MN, USA) and placed on a heated stage with flow chamber, and oxygenated RPMI without phenol red was heated and circulated through the chamber. The temperature of this medium was maintained at 36 ± 0.5 °C, as monitored by a probe immersed proximal to the sample. The microscope used was an FV1000MPE equipped with an XLPLN25XWMP Super 25 × 1.05-N.A. water immersion objective (Olympus, Tokyo, JPN). The excitation laser used was a 10-W Mai Tai HP DeepSee-OL (Spectra-Physics, Santa Clara, CA, USA). For experiments with only GFP+ cells, the laser was tuned to produce a wavelength of 910 nm; for experiments with GFP+ and CMTMR, it was tuned to 860 nm. PLNs were imaged ~170 μm deep (58 × 3 μm Z steps), acquiring one image/Z/minute. These images were processed with Imaris (Bitplane, Zurich, CHE) and MATLAB R2013b (MathWorks, Natick, MA, USA), as previously described [40]. Shown are maximal intensity Z-projections at a representative time point during the 30-minute imaging session.

### 2.15. Graphing and Statistical Analyses

Data visualization and statistical analyses were performed using Prism version 5 (GraphPad Software, San Diego, CA, USA). Throughout, asterisks used to denote *p*-values by indicated statistical test: * *p* ≤ 0.05, ** *p* ≤ 0.01, *** *p* ≤ 0.001.

## 3. Results

### 3.1. Light Chain Pairing with VH125 Determines Ig Affinity for Insulin

We began by determining the insulin-binding kinetics of multiple light-chain variable regions (VL) paired with the VH125 heavy chain. This included insulin-binding Ig 125, which is composed of VL125 combined with VH125, the functional equivalent to mAb125 [20]. Additionally, we generated a high-affinity anti-insulin Ig by immunizing VH125 transgenic B cells NOD animals with porcine insulin and screening multiple VLs cloned from responding B cells (data not shown). Of these, we selected a high-binding Ig, A12 (VLA12 + VH125), for further study. A lower-affinity Ig, EW6 (VLEW6 + VH125) was generated in an earlier study [25]. To reduce variability between these molecules, the Igs were created as chimeras in which the VL portions of the light chains were embedded in human kappa, and VH125 was embedded in human IgG1 heavy chain, as previously described [31].

**Figure 1:**
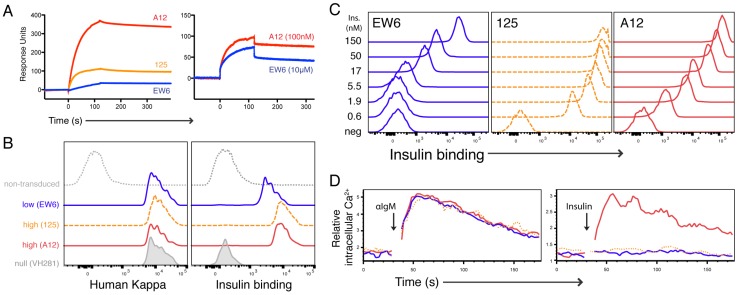
Light chain pairing with VH125 affects affinity for insulin. (**A**) SPR of recombinant Ig at 1 μM insulin concentration (left); comparison of high-affinity A12 binding 100 nM insulin and low-affinity EW6 binding 10 μM insulin (right); (**B**) VH125 transgenic bone marrow was transduced with light-chain-encoding retrovirus generating TR-B cells for analysis of BCR functionality in vitro. B220+, GFP+, IgM+ TR-BCR surface expression assessed by staining for human kappa constant region (left). Binding to labeled insulin (~50 nM) by A12, 125, and EW6 compared to GFP- and VH281 + A12 (right). (**C**) Binding equilibria titration performed using multiple dilutions of labeled insulin reveals 125 binds insulin more strongly than A12 when expressed as a BCR; (**D**) TR-B cell [Ca^2+^]_i_ response to stimulation with 5 μg/mL anti-IgM (left) or 50 μg/mL insulin (right): A12 (red), 125 (dashed orange), EW6 (blue). Data are representative of at least three individual experiments.

Recombinant Ig was produced by transient transfection of human endothelial kidney (HEK) 293 cells, and purified chimeric Ig was analyzed by surface plasmon resonance (SPR) for insulin-binding kinetics (Figure 1). For these studies, Ig was immobilized on the SPR chip surface and human insulin was injected in the fluid phase. In each experiment, analyses of association and dissociation kinetics were performed at multiple concentrations of soluble insulin. Shown here are representative response curves, illustrating the differences in insulin binding between Igs (Figure 1). The quantitative K_D_s were determined using a modified Langmuir isotherm model for association and dissociation rates, aggregated from multiple insulin dilutions and three independent experiments. A12 displayed the highest affinity for insulin (6.6 × 10^−9^ M), followed by 125 (1.6 × 10^−8^ M), and EW6 (3.8 × 10^−6^ M). Importantly, our experimental results were consistent with those previously reported for mAb125 of 3 × 10^−8^ M, validating this approach [20,23].

Having identified and characterized high- and low-affinity insulin-binding Igs, we began to test their function as BCRs. Our approach involved the expression of retroviral light chains in VH125 transgenic donor cells. We found that IL-7 bone marrow culture-derived immature pro-B cells were amenable to transduction, allowing the generation of model TR-B cells in vitro (Figure 1; Supplementary Materials, Figure S1). Retrogenic light chains were expressed on the cell surface and conferred insulin binding when paired with VH125, but not with the non-insulin-binding VH281 (Figure 1 and data not shown). Additionally, we determined that the epitope specificity was conserved between the VLs and soluble Ig and surface IgM (sIgM): using competitive inhibition of binding to labeled insulin, soluble 125 IgG blocked subsequent binding by the 125 BCR, as well as A12 and EW6 (Supplementary Materials, Figure S1). As we compare situations in which a variable number of receptors may be involved in binding, and valency becomes a variable, we will use the operative term “avidity”; when valency is presumed to be constant, or when discussing the theoretical bimolecular interaction of a single insulin molecule with a single immunoglobulin, we will use the term affinity.

Titration of biotinylated human insulin binding showed that EW6 has the lowest avidity for insulin when expressed as a BCR, corresponding to the lowest affinity by SPR (Figure 1). Surprisingly, 125 BCR appeared to bind insulin more strongly than A12. This finding was discordant with our SPR observations that A12 Ig has the highest affinity for insulin, and illustrates the importance of examining both soluble and membrane-bound Ig–Ag interactions.

To assess the signaling capability of retrogenic BCRs, relative intracellular Ca^2+^ ([Ca^2+^]_i_) was measured before and during stimulation by cross-linking BCR with polyclonal anti-IgM f(ab’)_2_ (Figure 1). All retrogenic BCRs mediated robust signaling following aggregation with this high-affinity anti-Ig antibody, confirming functionality. However, only A12 mediated a detectable Ca^2+^ flux in response to soluble insulin. This result is likely due to the high on-rate for the A12 Ig, as determined by SPR. Additionally, the ability of soluble insulin to induce detectable BCR signaling may be due to the formation of insulin dimers at micromolar concentrations, here stimulated with 9 µM human insulin (Figure 1).

**Figure 2:**
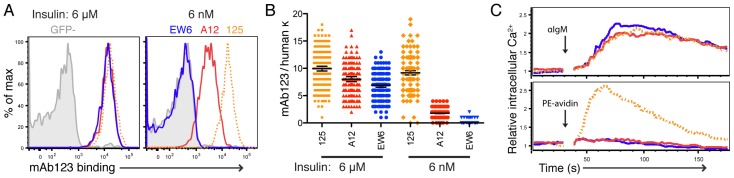
125 BCR has high avidity for insulin. (**A**) Receptor occupancy measured by mAb123 binding to TR-B cells (gated on B220+, GFP+, IgM+) incubated with 6 μM (left) or 6 nM insulin (right); (**B**) comparison of relative mAb123 binding to retrogenic light chain surface level on a per cell basis at 6 nM and at 6 μM. Each dot reflects the relative fluorescence of a single TR-B cell in both channels. (**C**) Calcium mobilization of A12 (red), 125 (dashed orange), and EW6 (blue) TR-B cells in response to stimulation with 5 μg/mL anti-IgM (top); calcium mobilization following pre-incubation with approximately 6 nM biotin-insulin and crosslinking with PE-avidin (bottom). Shown are representative results from at least three separate experiments for (**A**,**B**), and two separate experiments for (**C**).

Measurement of unlabeled insulin binding to BCR on B cells was crucial to our study of Ag acquisition. Previous studies have used mAb123 for this purpose: mAb123 is an anti-insulin Ab that recognizes an epitope on the B chain of insulin, which is spatially distinct from that recognized by mAb125, thus is not blocked when insulin is bound by 125 [20,24,39,41]. We validated this relationship in our model by incubating TR-B cells with varying concentrations of unlabeled human insulin, followed by probing with labeled mAb123 (Figure 2). Again, 125 displayed a high avidity for insulin, as demonstrated by receptor occupancy at low insulin concentrations: 125 TR-B cells retained 92 ± 2% of maximal occupancy at 6 nM insulin, whereas A12 occupancy was 22.2 ± 0.4% and EW6 was negligible (Figure 2).

To further confirm the ability of 125 BCR to bind more strongly than A12 at nanomolar concentrations of insulin, we pre-equilibrated cells with 6 nM biotin-insulin, cross-linked with PE-avidin and measured intracellular calcium mobilization. Cross-linking induced Ca^2+^ responses only in TR-B cells bearing 125 sIgM (Figure 2). We interpreted this as reflecting an increased retention of biotin-insulin on the surface of 125 TR-B cells, as compared to A12. To more quantitatively measure the off-rate, we also used cold-competitor disassociation experiments, in which we found that 125 TR-B cells exhibited a significantly longer half-life of insulin binding than A12 (Supplementary Materials, Figure S1).

Together, these results demonstrate that 125 exhibits very strong binding to insulin when expressed in sIgM form. This differs from the affinity of 125 IgG determined by SPR, which we found to be similar to previous reports. However, this high-avidity insulin binding by the 125 BCR in vitro is concordant with requirements for binding insulin in vivo, reconciling the difference between predicted and observed outcomes of previous studies.

### 3.2. VL CDR3 Determinants of Insulin Binding

**Figure 3:**
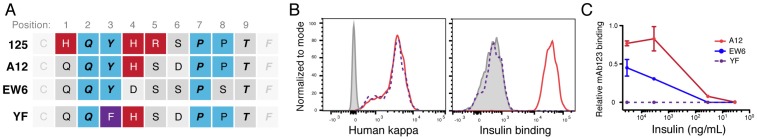
Light-chain CDR3 tyrosine is required for insulin binding by VH125 family antibodies. (**A**) Alignment of CDR3 regions of VL genes from framework region cysteine at position 0, highlighting positive-charged residues (red), conserved residues among these CDR3s (bold-italics), and the QYxxxPP motif (blue); (**B**) retroviral light-chain expression on VH125, GFP+, IgM+ cells transduced with A12 (red), YF (purple dashed), or non-transduced GFP- (gray-filled) (left). Binding to labeled insulin by A12 compared to YF mutant and non-transduced GFP- (right). (**C**) Detection of receptor occupancy at multiple concentrations of unlabeled insulin using mAb123 normalized to IgM level. Shown are data representative of at least three experiments.

The CDR3 regions of the insulin-binding light chains used for this study were derived from multiple germline genes, with VLA12 likely derived from V_Κ_ “4-57-1” and VLEW6 is similar to V_Κ_ “4-74” or “4-73”. These light-chain pairings have been implicated in insulin binding when paired with VH125 [41]. When these VL CDR3 sequences were aligned, certain amino acid residues were conserved between these insulin binding light chains (Figure 3). Of particular interest is a motif of QYxxxPP. The proline residue(s) light-chain CDR3s have been previously identified, and likely function in binding to insulin [23,41]. For this study, we chose to mutate the conserved position 3 (p3) tyrosine to assess the role of this hydroxyl group on insulin binding. The epitope of mAb125 has been mapped to the A-loop region of the insulin molecule [20]. The insulin A-loop has two hydroxyl-containing side chains that could form hydrogen bonds with the VL tyrosine, stabilizing the Ig–insulin binding.

To test the hypothesis that the light-chain CDR3 p3 tyrosine plays an important role in insulin binding, we created a mutant of the high-affinity VLA12, replacing the p3 tyrosine with phenylalanine. This light chain was named “VLYF,” and when paired with VH125 the resultant Ig is called “YF.” We found that the p3 tyrosine is required for insulin-binding activity by the high-affinity A12 BCR. This was demonstrated by its failure to bind labeled insulin and undetectable receptor occupancy with mAb123 following incubation with unlabeled insulin (Figure 3). These results demonstrate the shared requirement of heavy chain and light chain interactions with insulin for detectable binding.

### 3.3. Induction of Tolerance in Vitro Is Affected by Affinity of BCR for Insulin and Genetic Background of the TR-B Cell

It was previously demonstrated that culture of immature 125Tg B cells with insulin in vitro induces tolerance [22]. We used a similar approach, preparing immature TR-B cells in IL-7 culture, followed by addition of varying concentrations of human insulin. In early studies, we found that when immature high-affinity TR-B cells were cultured with insulin they likely underwent receptor editing, allowing for the expression of endogenous and retroviral light chain (data not shown). In the studies described here, we utilized RAG−/−.VH125 donor bone marrow cells, since they are incapable of receptor editing. To assess clonal deletion that might occur because the cells could not edit, we compared the percentage of GFP+ cells recovered following mock and insulin treatment in culture. In control experiments an approximate 20% decrease in GFP+ cells was seen in cultures treated with anti-IgM by 48 h, but only slight decreases (<10%) were observed upon treatment with high concentrations of insulin, suggesting that clonal deletion does not play a significant role in this model (Supplementary Materials, Figure S2).

**Figure 4:**
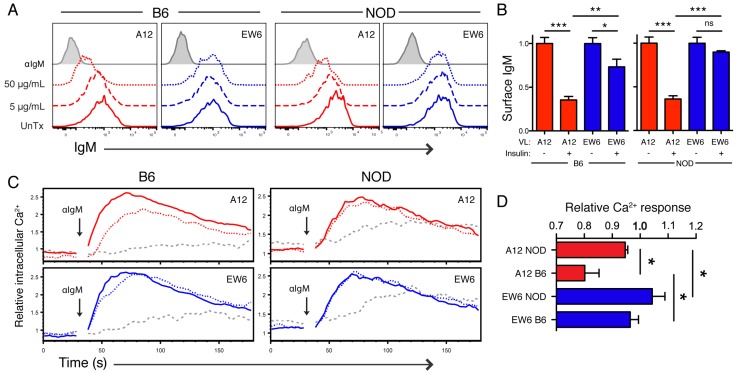
BCR affinity for insulin affects susceptibility to in vitro tolerance induction. (**A**) Surface IgM expression of high- and low-affinity TR-B cells from B6 or NOD background, gated on B220+ GFP+, 48 h post-treatment; (**B**) relative levels of B6 and NOD high-affinity TR-B surface IgM 48 h post-treatment with 50 µg/mL human insulin, normalized to untreated control; (**C**) comparison of calcium responses to stimulation with 5 μg/mL anti-IgM following culture with 50 μg/mL human insulin (dotted), anti-IgM (gray dashed), or untreated (solid) for 48 h; (**D**) relative Ca^2+^ response of TR-B cells as in (**C**), displayed as mean of area under the curve and peak height, normalized to untreated controls. Shown here are combined or representative results from more than three experiments, error bars denote SEM of replicates, Student *t*-test used (* *p* ≤ 0.05, ** *p* ≤ 0.01, *** *p* ≤ 0.001).

Two hallmarks of B cell tolerance include downregulation of surface IgM and reduced BCR signaling following stimulation. We found that culture of TR-B cells with high insulin concentrations (50 μg/mL) resulted in significant downregulation of high-affinity A12 BCR at 4, 24, and 48 h in both NOD and B6-derived cells (Figure 4, 48 h shown). Low-affinity EW6 retained high surface IgM levels in NOD TR-B cells, even at concentrations known to effectively saturate these receptors (Figure 1, Figure 2 and Figure 4). Interestingly, only B6 EW6 TR-B cells exhibited a significant down-modulation of BCR level at high concentrations of insulin, as compared to counterparts derived from NOD background.

The affinity of the BCR also affected relative Ca^2+^ mobilization following restimulation in TR-B cells cultured in high-insulin conditions. In B6 background, high-affinity A12 B cells showed a tendency toward tolerance, while EW6 did not, presumably due to its lower affinity. In cells derived from the NOD background, responses of both high and low affinity cells were not affected by preincubation with insulin (Figure 4). Together, these results demonstrate that genetic diabetes resistance contributes to tolerance in high-affinity immature insulin-binding B cells, which are more readily tolerized in vitro than their low-affinity counterparts, as measured by surface IgM level and calcium mobilization. This capacity for tolerance induction in B6 cells is compromised by the NOD genetic background.

### 3.4. Affinity-Dependent Tolerance Affects Autoantigen Presentation

**Figure 5:**
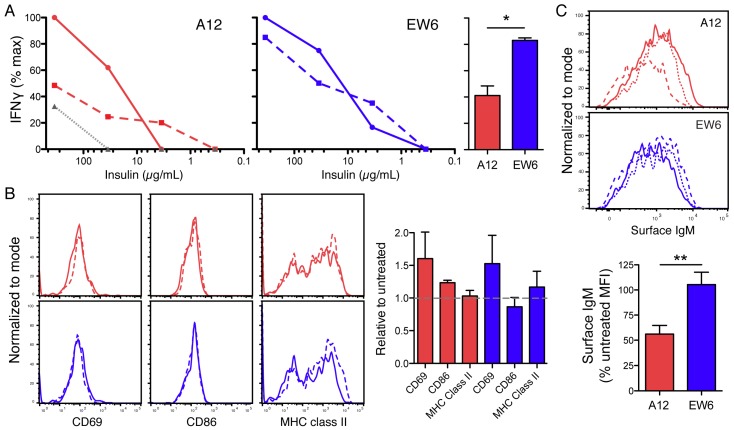
B cell tolerance affects ability to present antigen to T cells. (**A**) IFNγ secretion by PD12-4.4 T cell clones co-cultured with TR-B cells and indicated concentrations of insulin (solid lines) or following pre-treatment with 35 μg/mL of insulin for 48 h (dashed lines). Stimulation of PD12-4.4 T cells with PECs was only detectable at the highest insulin concentration (gray dotted lines). Bar graph of replicate cultures of tolerant TR-B cells restimulated with 350 μg/mL of insulin, normalized to untreated controls (right). (**B**) Activation marker expression on A12 immature TR-B cells (top), or EW6 (bottom), cultured with 35 μg/mL insulin for 48 h (solid), or without (dashed); bar graph of replicate cultures of A12 (red) or EW6 (blue) TR-B cells cultured with 35 μg/mL insulin for 48 h, normalized to untreated; (**C**) surface IgM staining of TR-B cells cultured with 35 μg/mL insulin for 2 days (dotted) or 4 days (dashed), as compared to untreated (solid); surface level of IgM on tolerized TR-B cells from input for antigen presentation to T cell clones, relative to untreated. Data representative of three independent experiments, error bars represent SEM, Student *t*-test used (* *p* < 0.05, ** *p* < 0.01).

Since a pathogenic function of autoreactive B cells in diabetes can be presentation of self Ag, we measured the capacity of these immature TR-B cells to present Ag to a T cell clone specific for insulin. This clone, PD12-4.4, secretes IFNγ upon TCR stimulation by insulin B9-23 peptide bound to H2^g7^ [42,43]. Due to the MHC class II requirement, NOD TR-B cells were tested as APCs. Naïve TR-B cells, or those previously incubated for 48 h in high insulin concentrations (35 μg/mL), were compared in their ability to stimulate IFNγ production by T cells upon addition of insulin. The naïve immature TR-B cells were potent stimulators of T cell clone PD12-4.4 activation, demonstrating their capacity to process and present antigens (Figure 5). However, TR-B cells were incapable of activating naïve insulin-specific BDC12-4.1 Tg T cells (data not shown). This limitation is likely due to the immature nature of the TR-B cells. We found that these IL-7 culture-derived cells do not upregulate activation and costimulatory molecules following stimulation (Figure 5), as expected from previous studies [44]. However, the PD12-4.4 T cell clones may be stimulated by the immature B cells because they are Ag-experienced [45].

A12 TR-B cells that were pre-incubated with insulin for 48 h had significantly reduced ability to activate a T cell response compared with the low-affinity EW6 cells (Figure 5). This impairment of T cell activation was not due to downregulation of costimulatory molecules or MHC class II: these levels were relatively unchanged in all conditions (Figure 5). As we had found in the tolerance experiments described above, surface BCR levels were significantly reduced at both two and four days post-insulin treatment (Figure 5). Indeed, we found that relative surface IgM level at time of co-culture with T cells correlated with subsequent IFNγ production (*p* = 0.017).

### 3.5. BCR Affinity Affects Autoantigen Acquisition in Vivo

Circulating insulin concentrations are significantly lower than those used in most of the above in vitro studies. Consequently, we hypothesized that only high-affinity TR-B cells would be capable of acquiring detectable insulin in vivo. To test this hypothesis, we first attempted to generate bone marrow chimeric Ig light-chain retrogenic VH125 animals, in a method analogous to that used for TCR retrogenics [46]. However, we had no success in this approach (data not shown), corroborating a recent publication demonstrating that traditional retrogenic techniques are incompatible with BCR Ig genes [47]. Since we had optimized the generation of TR-B cells using IL-7-containing bone marrow cultures, an adoptive transfer approach was used to study these cells in vivo.

**Figure 6:**
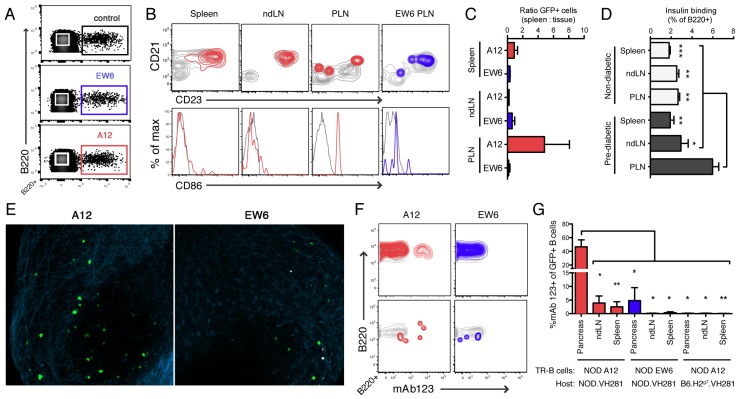
BCR affinity for insulin and host environment affects acquisition of autoantigen in vivo. (**A**) Adoptively transferred TR-B cells (GFP+) among B220+ splenocytes from NOD.VH281 recipients of A12, EW6, or VLA12+VH281 (control), one week post-transfer; (**B**) phenotypic comparison (CD21, CD23, and CD86) of TR-B cells (colored) to host VH281 (gray): A12 TR-B cells recovered from spleen, ndLN, PLN, as well as EW6 PLN; (**C**) ratio of TR-B cells (%GFP+) of total B cells in tissue vs. spleen; (**D**) comparison of insulin-binding B cells from non-diabetic NOD.VH125 females (blood glucose <120 mg/dL) or pre-diabetic (blood glucose 180-250 mg/dL); (**E**) 2-photon micrographs of PLNs from NOD.VH281 recipients of A12 (left) or EW6 (right) TR-B cells (GFP (green); second-harmonic generation of collagen, blue); (**F**) receptor occupancy detected by mAb123 staining of B220+ cells isolated from NOD.VH281 spleen (top) or PLN (bottom), comparing TR-B cells (colored) to host VH281 B cells (gray); (**G**) frequency of mAb123+ cells among adoptively transferred TR-B cells in NOD.VH281 or B6.H2^g7^.VH281 host animals. Data combined from five individual experiments, error bars represent SEM, Student *t* test used to test significance (* *p* < 0.05, ** *p* < 0.01).

The recipient animals were VH281 Tg females, thus the transferred TR-B cells would be the sole insulin-binding B cells present in recipients, following adoptive transfer. We assessed TR-B cells on day 4 and 7 post-transfer and found that a significant number of cells retained an immature phenotype on day 4 (Supplementary Materials, Figure S2). By day 7, the adoptively transferred GFP+ TR-B cells differentiated to mature follicular cells in the spleen (Figure 6). In the pancreas, tertiary lymphoid structures have been associated with insulitis observed in the progression of autoimmune diabetes, and may be enriched for islet antigen-reactive B cells [26,41]. Thus, we investigated the migration of TR-B cells to these sites of active inflammation, examining the islets and pancreatic lymph nodes (PLN) one week post-transfer. We found that TR-B cells were not concentrated in islets or non-draining lymph nodes (ndLN), but high-affinity A12 trend towards enrichment in the PLN, where they expressed CD86 (Figure 6).

The enrichment of high-affinity TR-B cells in the PLN is consistent with our observation that NOD.VH125 had an increased frequency of insulin-binding B cells corresponding to the early stages of diabetes (Figure 6). Young (6–8 weeks) non-diabetic female NOD.VH125 animals had a similar frequency of insulin-binding B cells in spleen, PLN, and ndLNs. However, we found with increased age the NOD.VH125 mice had a surge of insulin-binding B cells in the PLN, along with elevated blood glucose (180–250 mg/dL).

What is driving insulin-binding B cells to accumulate in the PLN? As discussed above, the B cells of the 125Tg NOD reportedly have high levels of receptor occupancy [11,13,24,41]. Using mAb123 staining immediately following isolation from various tissues, we assessed receptor occupancy of the TR-B cells in vivo. We found that BCR affinity for insulin determines the ability of the TR-B cell to acquire Ag. Low-affinity EW6 TR-B cells had nearly undetectable mAb123 binding, whereas high-affinity A12 TR-B cells exhibited significant levels of receptor occupancy in NOD tissues (Figure 6). In the spleen, A12 staining with mAb123 appeared bimodal, unlike reports of 125Tg cells that display relatively homogenous distribution of receptor occupancy (Figure 6) [24]. This heterogeneous staining was not due to a loss of A12 TR-B cells’ ability to bind Ag, as at seven days post-transfer splenic A12 TR-B cell binding of labeled insulin ex vivo is unimodal (Supplementary Materials, Figure S2).

In studies of 125Tg B6 animals, B cells are reported to be >90% receptor-occupied [13,24]. Using B6.H2^g7^.VH281 animals as hosts, we found that high-affinity A12 TR-B cells exhibited no detectable receptor occupancy in any tissue (Figure 6). It follows that we found low-affinity EW6 TR-B cells were also ignorant in B6.H2^g7^.VH281 animals: i.e., no mAb123+ EW6 cells were detected in any tissue (data not shown). Together, these findings demonstrate that BCR affinity, host, and tissue environment act in concert to allow for the acquisition and retention of the insulin autoantigen.

## 4. Discussion

B cells play an essential role in the etiology of diabetes. Currently, the consensus of the field is that their critical function is mediated by antigen presentation to T cells. However, this APC functionality is not due simply to the presence of B cells. The specificity of the BCR determines their diabetogenic activity: NOD mice with transgenic, innocuous B cells are protected from development of diabetes [11,12]. Conversely, mice with transgenic BCR biased towards insulin binding, or specific for insulin, develop diabetes [11,13].

VH125 heavy-chain-only transgenic mice—with their polyclonal repertoire—undergo more rapid and highly penetrant disease development than monoclonal 125Tg mice [11,25,26]. Thus we set out to examine and compare the functionality of B cells bearing receptors of higher and lower affinity for insulin than the 125Tg. Using SPR to test the affinity of multiple recombinant Ig sharing the VH125 heavy chain, we selected the insulin-binding A12 as the highest affinity, approximately 6.6 × 10^−9^ M; 125 was determined to be 1.6 × 10^−8^ M, and EW6 was 3.8 × 10^−6^ M. This represents an almost 3-log span of difference in affinity: with A12 over two times higher than 125, and EW6 hundreds or thousands-fold lower. These Ig molecules bind to overlapping or identical epitopes on the insulin molecule and are not polyreactive (Supplementary Materials, Figure S1). Studies of mAb125, which we extend to the VH125 family of Igs used here, have previously mapped the epitope to the A-loop of insulin and determined that this epitope is inaccessible on hormones bound to the insulin receptor [18,20].

We expressed these model Igs as sIgM to examine their functionality as BCR. Unexpectedly, 125 displayed a substantially higher avidity for insulin than A12 when expressed as a BCR. This observation was first based on 125 TR-B cells’ increased binding of labeled insulin in dose titration experiments (Figure 1), and receptor occupancy by unlabeled insulin (Figure 2). Additionally, we found that pre-equilibrating nanomolar biotinylated insulin, and subsequent crosslinking with avidin, caused a Ca^2+^ mobilization response in 125 TR-B cells only (Figure 2). It is possible that this high avidity is due to the increased number of positive-charged residues in the CDR3 region of VL125 (Figure 3), which have been implicated in insulin binding [21,48].

The difference between the observed avidities is peculiar when viewed in light of our SPR-determined affinities. It represents a dramatic reversal in the relative strength of insulin binding between these high-affinity Igs: A12 Ig had over 2-fold higher binding than 125 Ig by SPR, by cold competitor off-rate analysis 125 BCR was ~10-fold stronger than A12 BCR insulin binding (Supplementary Materials, Figure S1). All sIgM analyses use tight, consistent, gating on surface Ig expression, and are thus not likely due to differing receptor levels. The CDR3 for VL125 contains two histidine residues, which could coordinate a zinc ion with insulin B chain, representing a potential second epitope on monomeric insulin, increasing valency in the presence of zinc [17,49]. We attempted to examine this possibility by labeling TR-B cells with insulin-biotin in PBS + 2 mM EDTA (to chelate zinc), and found that the presence of a chelator did not affect the observed avidity of 125 sIgM (Supplementary Materials, Figure S1).

This represents an important finding in the context of reconciling previous studies of mAb125 and the 125Tg animal. The authors of these 125Tg studies noted that detecting BCR occupancy in vivo was unanticipated: due to the published K_D_ for mAb125 (30 nM) and circulating concentrations of insulin [11,20,24]. However, it would appear from our findings that 125 BCR exhibits orders of magnitude higher binding to insulin than its Ig form, used to determine affinity. From all accounts, it seems that 125 BCR-bearing B cells have an affinity for insulin suitable to binding the picomolar concentrations of circulating hormone in B6 and NOD animals, and this increased functional avidity serves as an explanation for the detected receptor occupancy and anergy-like phenotype of the 125Tg cells [13,24].

Comparison of the CDR3 regions of these insulin-binding VL genes revealed multiple conserved residues that may provide contacts or context required for this specificity. Both of the high-affinity CDR3s have p4 positive-charged residues, and a potential motif of QYxxxPP is highlighted (Figure 3). In previous analyses of light-chain CDR3s, the residues QYxxxPP are frequently identified, and the tandem prolines were significantly associated with binding to mouse insulin [23,41]. We hypothesized that the p3 tyrosine was required for binding insulin. To test this, we constructed a mutant of the high-affinity VLA12 gene, wherein the p3 tyrosine is replaced by phenylalanine (YF). This YF mutant light chain completely abrogated the insulin-binding ability of A12 (Figure 3), suggesting a requirement for this tyrosine residue in VH125-family Ig binding to insulin. As mentioned above, the epitope for A12 is likely the A-loop of insulin, which contains two hydroxyl side-chain residues; therefore, we inferred that the p3 tyrosine of the CDR3 may be forming hydrogen bonds with the A-loop of insulin.

After determining the insulin-binding characteristics of these Ig molecules, we began to explore the effect of BCR insulin-binding affinity on function. First, we utilized a model of in vitro tolerance induction in IL-7-derived immature TR-B cells. 125Tg B cells have been shown previously to be tolerized by incubation with insulin in this model [22]. We were curious about the effect of lower—perhaps more common—avidity anti-insulin BCRs. A12, which has nanomolar binding to insulin in both Ig and BCR forms, appears to have similar insulin binding to circulating anergic insulin-binding B cells we have previously described, though lacking polyreactivity (Supplementary Materials, Figure S1) [50].

Thus we selected A12 (high affinity) and EW6 (low affinity) to compare in functional studies, and found that A12 sIgM was significantly downregulated on both NOD and B6-derived TR-B cells when cultured with high insulin (Figure 4). This downregulation was BCR affinity-dependent, as A12 TR-B cells were significantly reduced compared to EW6. Additionally, A12 TR-B cells had reduced Ca^2+^ responsiveness to restimulation by anti-IgM following culture with insulin, as compared to their low-affinity counterparts.

Many previous studies have demonstrated defects in NOD B cells that lead to expanded autoreactivity or decreased tolerance as compared to diabetes-resistant genetic backgrounds [13,41,51,52,53,54,55]. Echoing this theme, we found that diabetes-resistant B6 TR-B cells were more readily tolerized than those derived from NOD in this system. This is illustrated by decreased surface IgM on B6-derived EW6, and by reductions in Ca^2+^ response in tolerized A12 cells from the B6 background (Figure 4). Interestingly, this effect in high-affinity cells occurs despite equivalent surface BCR downregulation: tolerized NOD A12 TR-B cells have ~40% BCR level, yet maintain similar response as controls. However, B6 A12 has significantly reduced Ca^2+^ influx (Figure 4). This is likely due to active inhibition of signaling, i.e., hyporesponsiveness of anergic B cells is not attributable to reduced expression of antigen receptors alone. We did not include standardization measures to determine absolute concentration. Note that the Ca^2+^ index reported is dependent on the shift of indo emission from ~470 nm (Ca^2+^-unbound) to ~400 nm (Ca^2+^-bound), which has a logarithmic relationship with intracellular concentration of Ca^2+^. As such, a reduction of ~50% of indo bound/free ratio may indicate a log reduction in intracellular Ca^2+^.

At the time of this communication, we have not successfully crossed B6.RAG2−/−.VH125 to the H2^g7^ MHC haplotype to compare NOD and B6 TR-B cell antigen presentation capability, or to allow transfer of B6-derived TR-B cells into NOD recipients. However, using NOD B cells as APCs, we observed a reduced ability to present antigen to insulin-specific T cells following previous tolerization (Figure 5). “Anergic” 125Tg cells have been shown to present antigen [13,56]; however, here we report that tolerized high-affinity anti-insulin B cells are capable APCs, but less effective than their naïve counterparts. In light of the increased tolerance imposed by B6 genetics, we predict that B6-derived high-affinity anti-insulin B cells may show even greater reductions in T cell priming, and are currently working to generate the genetic crosses necessary to test this hypothesis. As mentioned above, this APC function of insulin-specific B cells is likely their mechanism of action in driving diabetes. Previous work has mapped the primary diabetogenic T cell epitope to an insulin peptide of residues 9–23 of the B chain of mouse insulin 1 or 2 presented in I-A^g7^ [57]. More recently, hybrid insulin peptides were described as a novel class of diabetogenic T cell antigens [58]. The VH125-family Igs bind to an epitope on the insulin A-loop, which is conserved in both mouse insulin proteins and humans [20]; and are thus are not likely to share the identical peptide epitope as the T cell receptor. Instead, the high-affinity insulin-binding BCR could function to concentrate Ag in the endosome for processing and presentation on MHC.

To explore the function of high- and low-affinity TR-B cells in vivo, we used an adoptive transfer model, in which TR-B cells were sorted and transferred to VH281 hosts. Thus, the GFP+ cells will be the only insulin-binding B cells in the animal. We assayed the TR-B cells at days 4 and 7 post-transfer, and found that they appear mature and follicular in spleen and lymph node by day 7 (Figure 6 and data not shown). At one week post-transfer, GFP+ cells were detectable in the pancreatic lymph node (PLN) (Figure 6; Supplementary Materials, Figure S2). In NOD mice that received high-affinity A12 TR-B cells, we found the bulk of receptor-occupied cells were in the PLN or islets (Figure 6). The A12 cells in the spleen had detectable receptor occupancy, with a bimodal distribution (Figure 6). This staining pattern was not due to a lack of insulin-binding capacity (Supplementary Materials, Figure S2), and is likely an effect of location: either the insulin-bound cells are situated in the spleen in an area of increased Ag concentration, or are recirculating through the spleen having previously been to an area of high Ag concentration, i.e., the pancreas. Interestingly, a recent study which included co-adoptive transfer of class switch-competent knock-in VH125 B cells with insulin-specific T cells found a similar distribution of insulin-binding B cells enriched in the PLN as compared to the ndLN or spleen [59].

In NOD mice, only the low-affinity EW6 cells in the pancreas retained detectable BCR bound insulin—perhaps due to significantly higher Ag concentration or valency at this site. These results support a model in which bona fide low-affinity anti-insulin B cells in peripheral lymphoid organs are ignorant of insulin in their surroundings. Further, they display no increased frequency in the PLN as well as limited/no upregulation of CD86 (Figure 6). Thus, we propose that micromolar affinity insulin-binding B cells are not receptor occupied in peripheral blood or spleen, even in diabetes-susceptible NOD animals. An avidity threshold exists for detectable receptor occupancy and function in these compartments: EW6 falls below it, and A12 is sufficient in NOD to sense and respond to insulin in the PLN.

However, high-affinity A12 cells are clonally ignorant in B6-background animals (Figure 6). We observed no detectable mAb123 binding in A12 or EW6 cells in any tissue assayed: spleen, blood, PLN, ndLN (Figure 6; Supplementary Materials, Figure S2). These findings are in stark contrast to the observations of the 125Tg mouse, in which virtually all B cells are bound to insulin, likely from “cradle to grave” [41]. Our finding that B cells bearing non-polyreactive A12 BCR with nanomolar affinity for insulin appear clonally ignorant in B6 are in keeping with predictions for binding the hormone at picomolar circulating concentrations. Furthermore, this highlights an undescribed difference in the availability of insulin for B cell binding in NOD as compared to B6.

We propose that the difference between the previous findings in the 125Tg animals and this report is due to the incredibly high avidity of 125 BCR for insulin, which binds much more strongly than determined by studies of soluble Ig form. The A12 Ig, while having higher affinity for insulin by SPR, has a seemingly concordant BCR avidity, and thus illuminates a difference in the NOD pancreas environment that allows for autoantigen acquisition by B cells, as compared to the same cells in the B6 animal. Additionally, in keeping with reports highlighting decreases in B cell-intrinsic tolerance in NOD, we demonstrate that TR-B cells with high and low affinity for insulin are less susceptible to tolerance induction, as measured by inhibition of Ca^2+^ response and BCR downregulation (Figure 4).

Taken together, this study finds that a unique aspect of the NOD pancreas environment allows for the acquisition of insulin by B cells with sufficient affinity BCR. These B cells can be found in the PLN, where we suggest they can efficiently present antigen to insulin-specific T cells. Additionally, NOD B cells display decreased tolerance following antigen recognition. We propose that these findings highlight a trifecta that determines diabetes susceptibility: BCR affinity, autoantigen availability, and inadequate tolerance.

## Supplementary Materials

The following are available online at http://www.mdpi.com/2077-0383/5/11/98/s1, Figure S1: Extended characterization of model Igs. Figure S2: Tolerance and development of TR-B cells.
