# Supplementary material for: B Cell Receptor Affinity for Insulin Dictates Autoantigen Acquisition and B Cell Functionality in Autoimmune Diabetes
Source: J Clin Med. 2016 Nov 8;5(11):98. doi: 10.3390/jcm5110098 (PMC5126795; doi:10.3390/jcm5110098)
Supplement: Supplementary file 1 [file jcm-05-00098-s001.docx]

**Supplementary Materials: B Cell Receptor Affinity for Insulin Dictates Autoantigen Acquisition and B Cell Functionality in Autoimmune Diabetes**

Thomas A. Packard, Mia J. Smith, Francis J. Conrad, Sara A. Johnson, Andrew Getahun, Robin S. Lindsay, Rochelle M. Hinman, Rachel S. Friedman, James W. Thomas, and John C. Cambier

# 1. Supplemental Methods

1.1. Flow Cytometric Analysis of TR-B cells

Immature TR-B cells were prepared as described in methods: by retroviral transduction of IL-7 cultured bone marrow cells from transgenic donor animals. Cells were stained and analyzed by flow cytometry as described within.

1.2. 2D Off-Rate Analysis by Cold Competition

TR-B cells were prepared as described above, and allowed to equilibrate with biotin-insulin (1µg/mL for 30 min) on wet ice. Unlabeled insulin was subsequently added at 100 µg/mL, starting at 30 min prior to wash, then 15, 5, etc. At time 0, all cultures were quick washed as described in methods; and retained biotin-insulin was revealed with Brilliant Violet

421-streptavidin (0.2µg/mL, Biolegend). Along with the secondary, the described mixture of staining Abs for B220, IgM, and human kappa were added.

1.3. Ig-Mediated Cross-Inhibition of Insulin Binding

Recombinant Igs were prepared as described within. Biotin-insulin was prepared as within, and aliquots of biotin-insulin were pre-incubated at a ~1:10 molar ratio with individual recombinant Igs or control human Ig (e.g. 1 µg insulin + 30 µg 125 Ig). These mixtures were then used to label TR-B cells, prepared as above, except the labeling Ab cocktail was added following washing, concurrent with addition of the secondary.

1.4. Insulin and Polyreactive ELISA

Polystyrene plates (Nunc) were coated with 10 µg/mL recombinant human insulin (Sigma) or LPS (Sigma) in PBS overnight at 4°C in a humidity-controlled chamber. Chromatin was obtained from Dr. Larry Wysocki (National Jewish Health), and coated at a dilution empirically determined to be optimal for ELISA (1:100) in PBS overnight at 4°C in a humidity-controlled chamber. Coated wells were blocked with 0.5% BSA (Sigma) in PBS for one hour at room temperature.

Recombinant Igs, prepared as described above, were compared to those prepared as part of the study described in Smith, et al. [1]. Briefly, Igs were diluted in blocking buffer, added to plate, and incubated at room temperature for two hours. Bound Ig was detected using HRP anti-human IgG (Bio-Rad), and OptEIA TMB (BD Biosciences) as substrate. The reaction was quenched with 1N H3PO4 and optical density was measured at 450 nm.

1.5. Effect of zinc on insulin binding to TR-B cells

TR-B cells were prepared as above and before staining were washed and resuspended in PBS + 2 mM EDTA (Sigma). Biotin-insulin was added at the indicated approximate concentrations (4-30 nM), along with staining Abs as described within, and cells were incubated on ice for 30 minutes. After incubation cells were washed and incubated with Brilliant Violet 421-streptavidin (0.2 µg/ml, Biolegend) for 30 min on ice, followed by washing and analysis by flow cytometry.


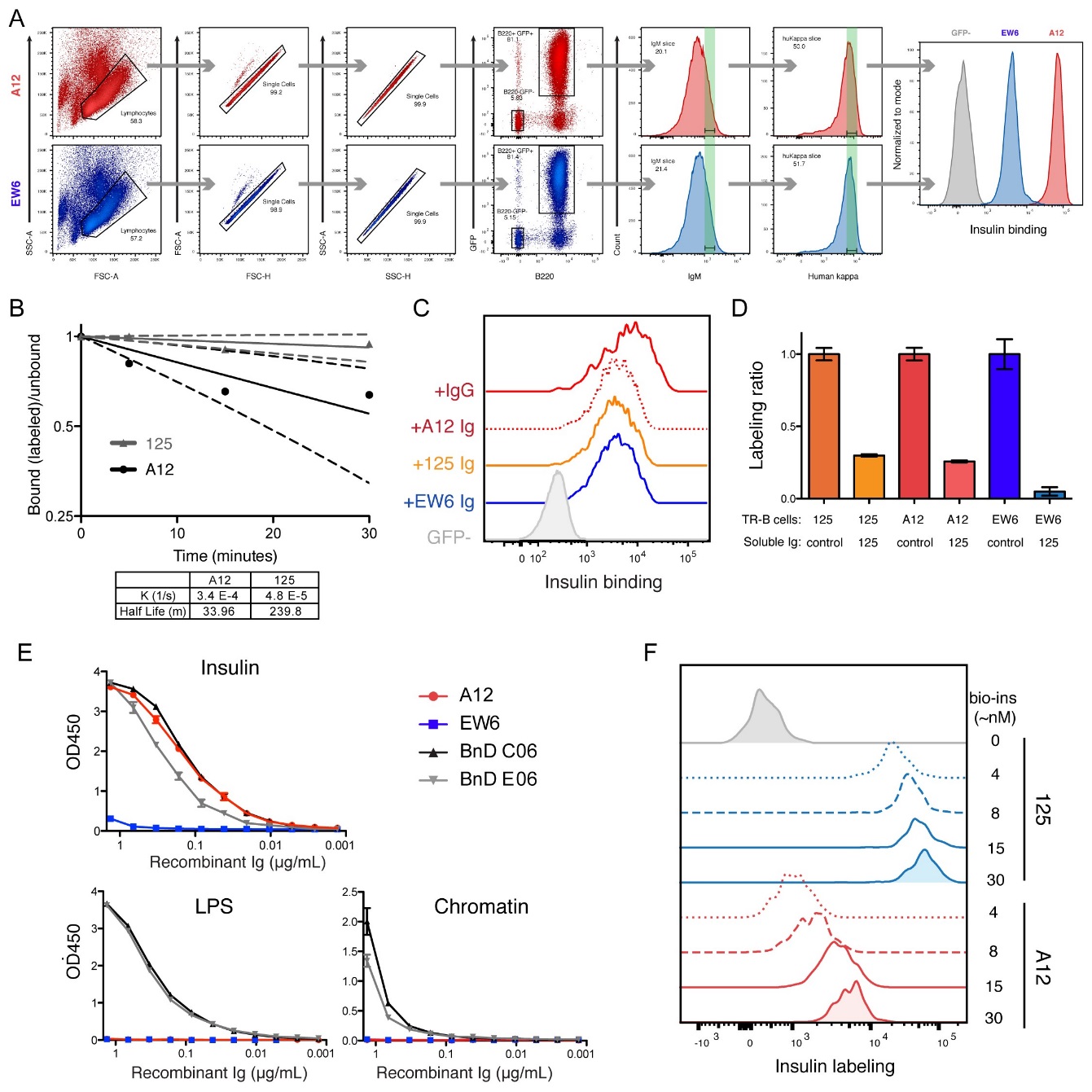


**Figure S1. Extended characterization of model Igs.** (**A**) Examples of gating of TR-B cells as performed within the main text: gating on singlet lymphocytes, B220+, GFP+, IgM+, human kappa+, reveals insulin-binding TR-B cells. (**B**) Cold-competitor off-rate analysis of 125 and A12 BCR shows that 125 exhibits a slower off-rate of bound labeled insulin than A12. (**C**) Pre-incubation of biotin-insulin with soluble insulin-binding Igs reduces subsequent capture by A12 TR-B cells, and (**D**) comparison of the binding to biotin-insulin by TR-B cells in the presence of control IgG or soluble 125 Ig, demonstrate steric competition at the Ag-Ig interface. (**E**) A12 and EW6 Igs are not polyreactive: A12 (red), EW6 (blue), BNDC06 (black), and BNDE06 (gray) recombinant Ig were assayed for their ability to bind the indicated Ag-coated plates by ELISA. (**F**) Labeling TR-B cells with insulin-biotin (at indicated concentrations) in PBS + 2 mM EDTA shows 125 maintains high avidity in presence of chelating agent.


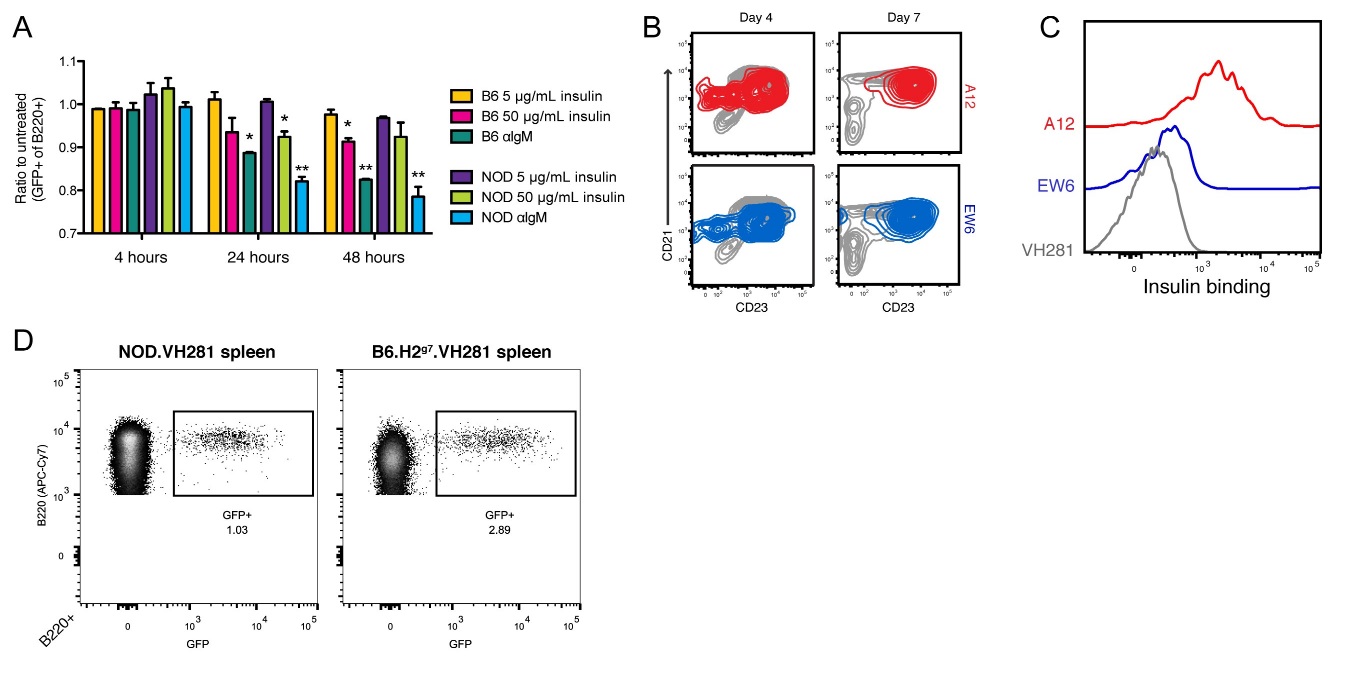


**Figure S2. Tolerance and development of TR-B cells.** (**A**) Analysis of clonal deletion by comparison of GFP+ fraction in treated vs. untreated control cells. Shown are high affinity A12 TR-B cells treated with 5 or 50 µg/mL human insulin, or 5 µg/mL goat-anti mouse IgM for indicated time. Each experimental condition is normalized to respective untreated control, and compared to this control by Student *t* test for significance (*p* = * < 0.05, ** < 0.01); (**B**) Splenic TR-B cells exhibit a mature-follicular phenotype one week post-transfer. Comparison of CD21 and CD23 expression of A12 and EW6 TR-B cells days four and seven post-transfer, gated on GFP+ B220+ cells or host VH281 B220+ GFP− cells (gray). (**C**) TR-B cells maintain insulin-binding capability following adoptive transfer. Day 7 post-transfer of TR-B cells, splenocytes were harvested and labeled *ex vivo* with biotin-insulin. GFP+ B220+ A12 and EW6 TR-B cells compared to host VH281 B220+ GFP−. (**D**) NOD.VH125.RAG−/−-derived TR-B cells can be adoptively transferred to MHC-matched B6.H2g7.VH281 recipients, and are recovered at equal or higher numbers following one week post-transfer (shown here are GFP+ A12 TR-B cells in the spleen).

References

1. Chung, J.B.; Wells, A.D.; Adler, S.; Jacob, A.; Turka, L.A.; Monroe, J.G. Incomplete activation of CD4 T cells by antigen-presenting transitional immature B cells: Implications for peripheral B and T cell responsiveness. *J. Immunol.* **2003**, *171*, 1758–1767.
